# X-ray propagation through a kinoform lens

**DOI:** 10.1107/S1600577522008244

**Published:** 2022-09-29

**Authors:** Weihong Sun, Yong Wang, Xiangyu Meng, Junchao Ren, Jiefeng Cao, Junqin Li, Renzhong Tai

**Affiliations:** a Shanghai Institute of Applied Physics, Chinese Academy of Sciences, Jialuo Road 2019, Jiading District, Shanghai 201800, People’s Republic of China; b University of Chinese Academy of Sciences, Yuquan Road 19, Shijingshan District, Beijing 100049, People’s Republic of China; c Shanghai Advanced Research Institute, Chinese Academy of Sciences, Zhangheng Road 239, Pudong District, Shanghai 201204, People’s Republic of China; Uppsala University, Sweden

**Keywords:** kinoform lens, synchrotron radiation, geometrical optics, wave optics

## Abstract

Combining geometric ray tracing and wave optics propagation, a new simulation model named LWF is established to calculate the full coherent X-ray propagation through a kinoform lens. The LWF model is used to analyze the X-ray propagation through long and short kinoform lenses and calculate the intensity distribution at the focal plane. When the aperture is large, the focal spot for the long kinoform lens is smaller than that for the short kinoform lens. Due to the use of the geometric ray-tracing method to calculate the beam propagation inside the kinoform lens, the LWF model takes a low number of transversal wavefront segments, *i.e.* a short time, to achieve high accuracy. The simulation times for the one-dimensional and two-dimensional LWF models are 0.025 s and 5.3 s, respectively, with a calculation error of less than 0.5%. The high efficiency and high accuracy make the LWF model a strong tool in designing kinoform lenses.

## Abstract 2

A new model named LWF is established to quickly calculate X-ray propagation through a kinoform lens.

## 1. Introduction

Refractive focusing optical elements are widely used in hard X-ray submicrometre focusing due to their high working energy and flexible capability (Schroer *et al.*, 2005 ▸; Guilherme *et al.*, 2012 ▸; Evans-Lutterodt *et al.*, 2007 ▸). However, conventional X-ray refractive focusing optical elements (*e.g.* compound refractive lenses) have the disadvantage of strong X-ray absorption at their edges, and they cannot increase the photon flux and focusing capability by increasing the geometric aperture (Chen *et al.*, 2014 ▸; Schroer & Lengeler, 2005 ▸). On the basis of plano-concave lenses, the kinoform lens cuts off material with a path length of an integer multiple of X-ray wavelengths, which effectively increases the geometric aperture (Aristov *et al.*, 2000 ▸; Nöhammer *et al.*, 2003 ▸). Several models have been developed to optimize the design of kinoform lenses. The thin-lens approximation method (Buralli *et al.*, 1989 ▸) is a simple model with low calculation accuracy (Yan, 2010 ▸). The Takagi–Taupin description (TTD) of X-ray dynamical diffraction theory (Yan *et al.*, 2007 ▸) is a rigorously accurate method that is suitable for analysis of short kinoform lenses. However, the calculation time of the TTD method is affected by the length of the kinoform lens. Due to its long length with a step distribution along the optical axis, the long kinoform lens is not suitable for analysis by the TTD method (Yan, 2010 ▸). The beam propagation method (BPM) is a wave-propagation-based modeling approach (VanRoey *et al.*, 1981 ▸). Here, the kinoform lens is divided into a large number of slices along the optical axis, and the beam propagation through the uniform medium of a slice is calculated using angular spectrum theory (Goodman, 2015 ▸). The phase difference caused by the difference of the sliced medium is added to the complex amplitude. The BPM model has high calculation accuracy, but has low calculation efficiency due to the large number of slices (Yan, 2010 ▸). The design of a kinoform lens urgently needs a model with high efficiency and high accuracy.

This paper presents a kinoform lens simulation model, the ladder wavefront (LWF), which uses geometric optics to describe the propagation inside the kinoform lens and wave optics to calculate the propagation through free space. The LWF model has the advantage of both high accuracy and high efficiency. In this paper, the LWF model is used to simulate the propagation of fully coherent X-rays through long and short kinoform lenses and acquire the intensity distribution in the focal plane. The LWF model is benchmarked against the BPM model to confirm the model calculation accuracy. The accuracy and efficiency of the LWF model can be balanced by simply changing the number of elements within the wavefront.

## 2. LWF model establishment

The refractive index of the medium of a plano-concave lens is defined as *n* = 1 − δ + *i*β. On the basis of the plano-concave lens, the kinoform lens cuts off material with a path length of an integer multiple of X-ray wavelengths. The kinoform lens not only maintains the capability of submicrometre focus but also reduces X-ray attenuation. The step length *l* = *m*λ/δ generates an *m* integer-2π phase difference between X-ray propagation inside and outside the step, where λ is the wavelength and δ is the refractive index of the medium. The curved surface of the kinoform lens is elliptical and can be expressed as (Evans-Lutterodt *et al.*, 2003 ▸)

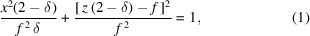

where *x* is the transverse coordinate of the lens, *z* is the coordinate along the optical axis, and *f* is the focal length. Since the long kinoform lens is widely applied in hard X-ray submicrometre focusing, we take the long kinoform lens as an example to introduce the LWF model.

**Figure 1:**
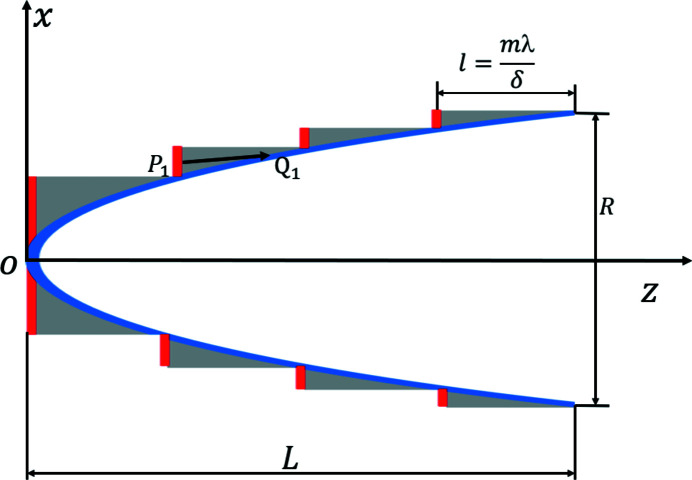
Schematic diagram of a long kinoform lens. The *x* and *z* axes denote the transversal and longitudinal directions, *L* denotes the total length of the lens, *R* denotes the aperture of the lens, *l* denotes the length of each step, and *P*_1_ and *Q*_1_ denote the points at the incident and exit surfaces, respectively.

The incident plane (red lines) is defined as the upstream surfaces of the kinoform lens, as shown in Fig. 1 ▸. The exit plane (blue curve) is defined as the downstream curved surface of the kinoform lens. The incident and exit planes are described as the *P* and *Q* planes, respectively. The geometric ray-tracing method is used to analyze the wavefront propagation through the incident surface to the exit surface. This method is suitable for both one-dimensional and two-dimensional conditions. The wavefront at the incident plane is divided into many small surface elements. The rays travel along the direction defined by the phase gradient within each element at the incident plane. Following Fermat’s principle (Born & Wolf, 1986 ▸), the rays enter the kinoform lens medium, transmit through the medium, and hit the exit plane. The path length caused by the ray traveling inside the kinoform lens is used to describe the phase change. Assume that a certain ray enters the medium from point *P*_1_ at the incident surface and hits point *Q*_1_ at the exit surface. *u*(*P*_1_) is the complex amplitude at point *P*_1_, and the complex amplitude *u*(*Q*_1_) at point *Q*_1_ can be expressed as

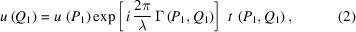




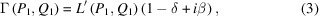




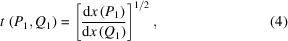

where *L*′(*P*_1_, *Q*_1_) is the distance from *P*_1_ to *Q*_1_; Γ(*P*_1_, *Q*_1_) is the corresponding path length describing X-ray attenuation and phase change; *t*(*P*_1_, *Q*_1_) is the complex amplitude transmission function, and *x*(*P*_1_) and *x*(*Q*_1_) are the coordinates of *P*_1_ and *Q*_1_. The kinoform lens is used as one of the focusing optical elements. For synchrotron radiation beamlines, the point source is generally located tens of metres upstream. The wavefront at the incident surface of the kinoform lens is not a plane wave but a divergent wave. Therefore, some rays can escape from the step edge of the kinoform lens. For simplicity, the escaped rays are ignored in the subsequent propagation. The total lost flux in the whole kinoform is less than 0.1%. Therefore, the LWF model still has high accuracy.

The X-ray propagation from upstream source plane *S* to incident plane *P* is simulated using wave optics. The propagation of X-rays through free space is based on the Huygens–Fresnel principle. For one-dimensional cases, the propagation formula is given as follows (Born & Wolf, 1986 ▸),

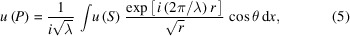

where *u*(*P*) and *u*(*S*) are the complex amplitudes at the source and incident planes; *r* is the propagation distance, and θ is the angle between the wavevector and the optical axis. For the two-dimensional case, the formula is described as follows (Born & Wolf, 1986 ▸),

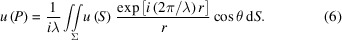

The meaning of each symbol is the same as for formula (5). The simulation method calculating the X-ray propagation from the exit plane to the focal plane is the same as that from the source to the incident plane.

**Figure 2:**
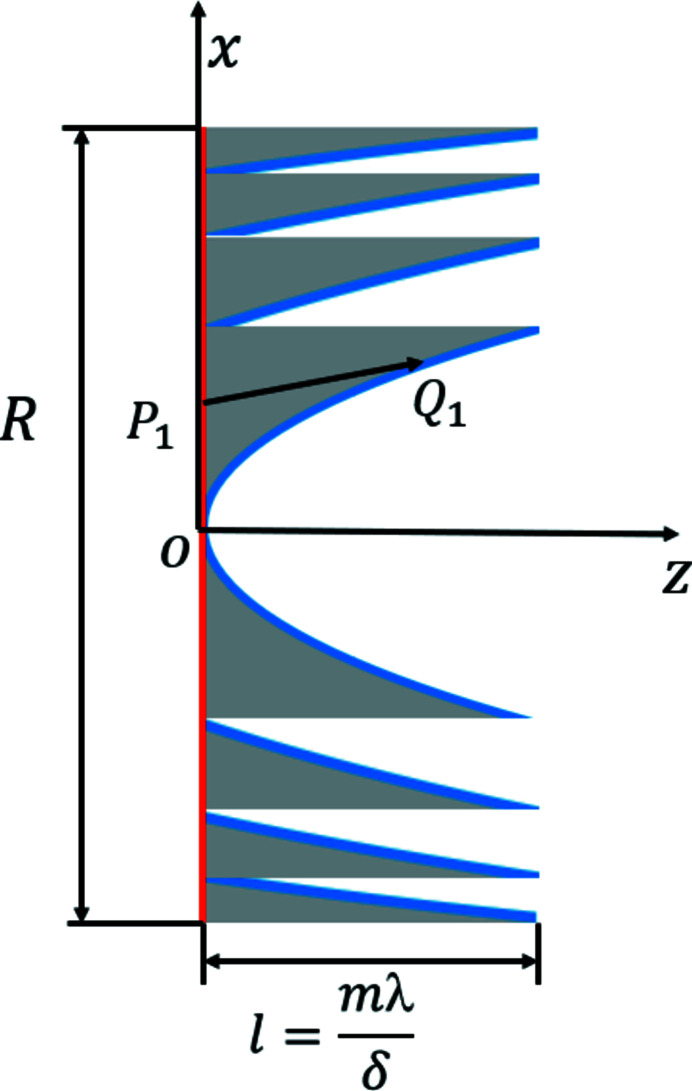
Schematic diagram of a short kinoform lens. The *x* and *z* axes denote the transversal and longitudinal directions, respectively, *R* denotes the aperture of the lens, *l* denotes the total length of the lens, and *P*_1_ and *Q*_1_ denote the points at the incident and exit surfaces, respectively.

Unlike the long kinoform lens, the steps for the short kinoform lens are designed for one plane, as shown in Fig. 2 ▸. The thickness of the short kinoform lens is *l* = *m*λ/δ. The left plane (red line) is the incident plane, and the right curved surface (blue line) is the exit surface. The exit surface function of different steps for the short kinoform lens is always hyperbolic, while the surface function for the long kinoform is elliptical. The formula for the short kinoform lens can be expressed as follows (Liao *et al.*, 2015 ▸),

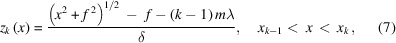

where *k* is the step number. The wavefront propagation through the short kinoform lens is also defined by formula (2).

## 3. Simulation results

### 3.1. Simulations for one-dimensional long kinoform lens

**Figure 3:**
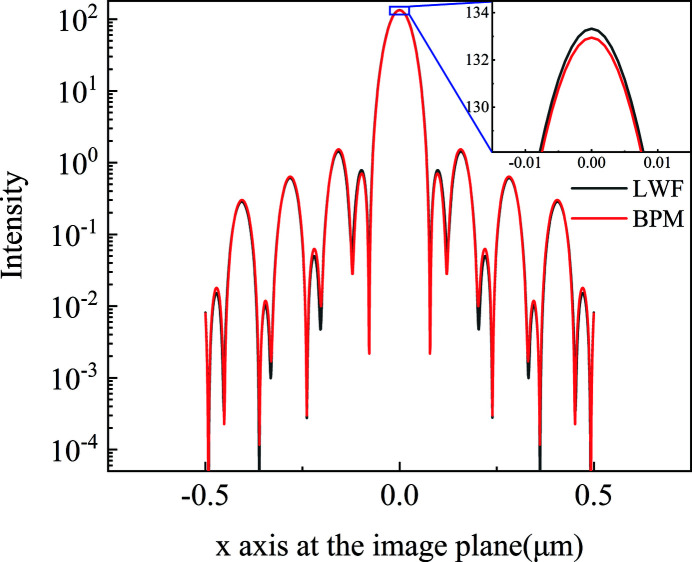
The intensity distribution at the focal plane for the one-dimensional long kinoform lens calculated from the LWF model (black line) and BPM model (red line). The spot size r.m.s. is 26.1 nm.

The LWF model is used to simulate wavefront propagation through a one-dimensional long kinoform lens. The source energy is 11.3 keV; the light intensity has a Gaussian distribution with r.m.s. size of σ = 7 µm, and the complex amplitude is a plane wave. The kinoform lens is located 20 m downstream of the light source. The aperture of the kinoform lens is 79.8 µm; the total length of the lens is 4.594 mm with each step length of 229.7 µm, and the step number of the lens is 20. The material of the lens is silicon. When the energy is 11.3 keV, δ and β are 3.8214 × 10^−6^ and 4.5613 × 10^−8^, respectively. The focal length of the lens is 50 mm. The distance between the lens exit plane and focal plane is 45.51 mm. The incident surface is divided into 400 elements with each element of size 199.5 nm. Fig. 3 ▸ shows the intensity distribution in the focal plane. The focus spot size r.m.s. calculated from the LWF model (black line) is 26.1 nm. The intensity profiles have apparent diffraction peaks with a period of 60 nm, which are determined by the finite size of the kinoform lens aperture and the periodical step structure. The LWF model is used to benchmark against the BPM model. For the BPM model, the transversal wavefront is divided into 7000 elements of 11.4 nm. The number of wavefront slices perpendicular to the optical axis is 5000, with each slice having a thickness of 0.91 µm. The intensity distribution from the BPM model (red line) is similar to the LWF result, and the difference between the maximum intensities is less than 0.5%. The LWF model uses ray tracing and the BPM uses angular spectrum theory to simulate wavefront propagation from the kinoform lens incident to the exit planes. Ray tracing and angular spectrum theory are both suitable for describing X-ray propagation through an inhomogeneous medium. The difference in the calculation result between LWF and BPM is quite small; possible reasons are that ray tracing relates to geometry optics and angular spectrum theory to wave optics.

**Figure 4:**
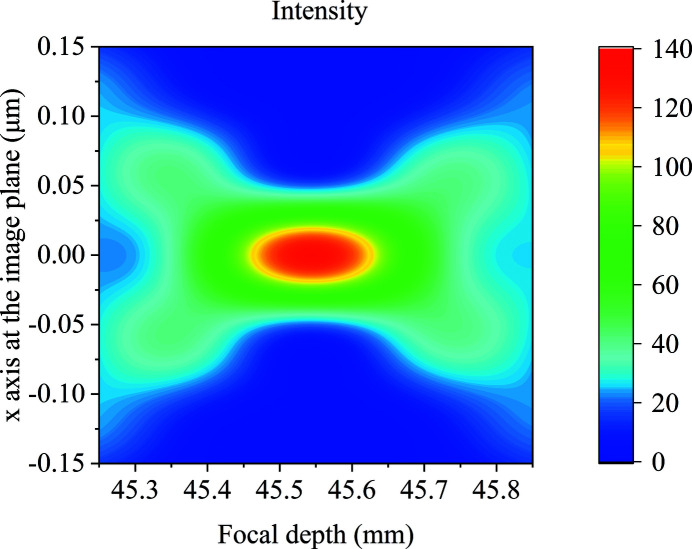
The intensity distribution along the focus depth for the one-dimensional long kinoform lens. The zero position is defined at the back plane of the kinoform lens.

The LWF model has high calculation efficiency. It can be used to calculate the intensity distribution along the focus depth, as shown in Fig. 4 ▸. The focus depth is 287.9 µm. There is a 129 µm position difference between the simulated and theoretical focal points due to the limited source size and constant phase distribution at the source plane, which also embodies the importance of high calculation efficiency.

### 3.2. Simulation for one-dimensional short kinoform lens

**Figure 5:**
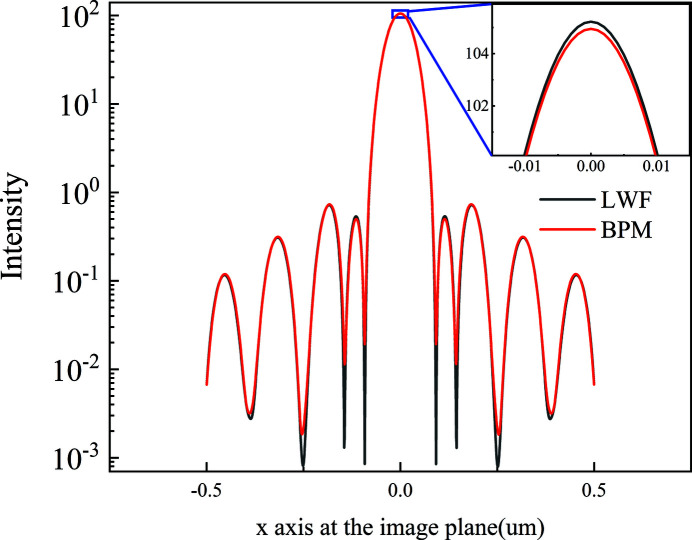
The intensity distribution at the focal plane for the one-dimensional short kinoform lens calculated from the LWF model (black line) and BPM model (red line). The spot size r.m.s. is 30.0 nm.

The LWF model is used to simulate wavefront propagation through a one-dimensional short kinoform lens. The source parameters and short kinoform lens parameters except the total length are the same as given in Section 3.1. The short kinoform lens length is 229.7 µm. The distance between the lens exit plane and focal plane is 49.99 mm. The incident surface is divided into 400 elements with each element of size 199.5 nm. Fig. 5 ▸ shows the intensity distribution in the focal plane. The focus spot size r.m.s. calculated from the LWF model (black line) is 30 nm. The LWF model is used to benchmark against the BPM model. For the BPM model, the transversal wavefront is divided into 7000 elements of 11.4 nm. The number of wavefront slices perpendicular to the optical axis is 5000 with each slice of thickness 45.9 nm. The intensity distribution from the BPM model (red line) is similar to the LWF result, and the difference between the maximum intensities is less than 0.5%. The intensity profiles have apparent diffraction peaks, which are determined by the finite size of the kinoform lens aperture and the periodic step structure. The focus spot size r.m.s. of the short kinoform lens calculated from the LWF model is 30 nm, which is larger than the focus size r.m.s. of the long kinoform lens. The surfaces of the long kinoform lens and the short kinoform lens are elliptical and hyperbolic, respectively. The closer to the aperture edge, the greater the difference between the wavevector refraction by the two surfaces. With increasing aperture of the kinoform lens, the focusing ability of the short kinoform lens is reduced.

**Figure 6:**
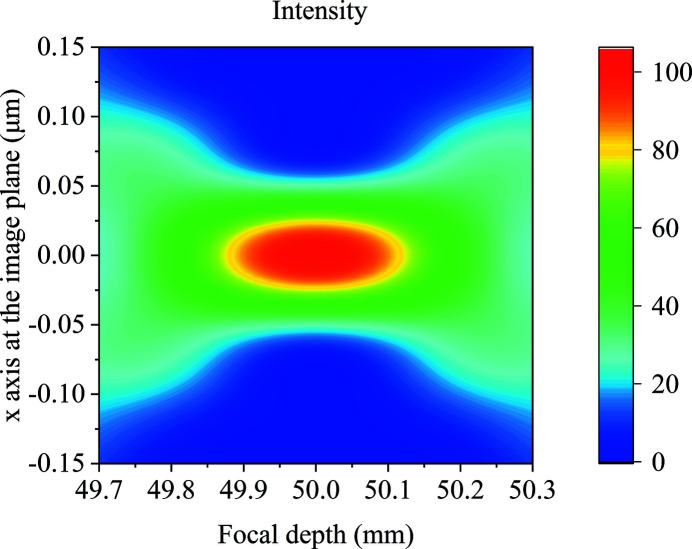
The intensity distribution along the focus depth for the one-dimensional short kinoform lens. The zero position is defined at the back plane of the kinoform lens.

The LWF model has high calculation efficiency. It can be used to calculate the intensity distribution along the focus depth, as shown in Fig. 6 ▸. The focus depth is 360 µm, which is slightly larger than the focal depth of the long kinoform lens. This is because the short kinoform lens is hyperbolic, which reduces the focusing capability of the kinoform lens.

### 3.3. Simulation for two-dimensional long kinoform lens

**Figure 7:**
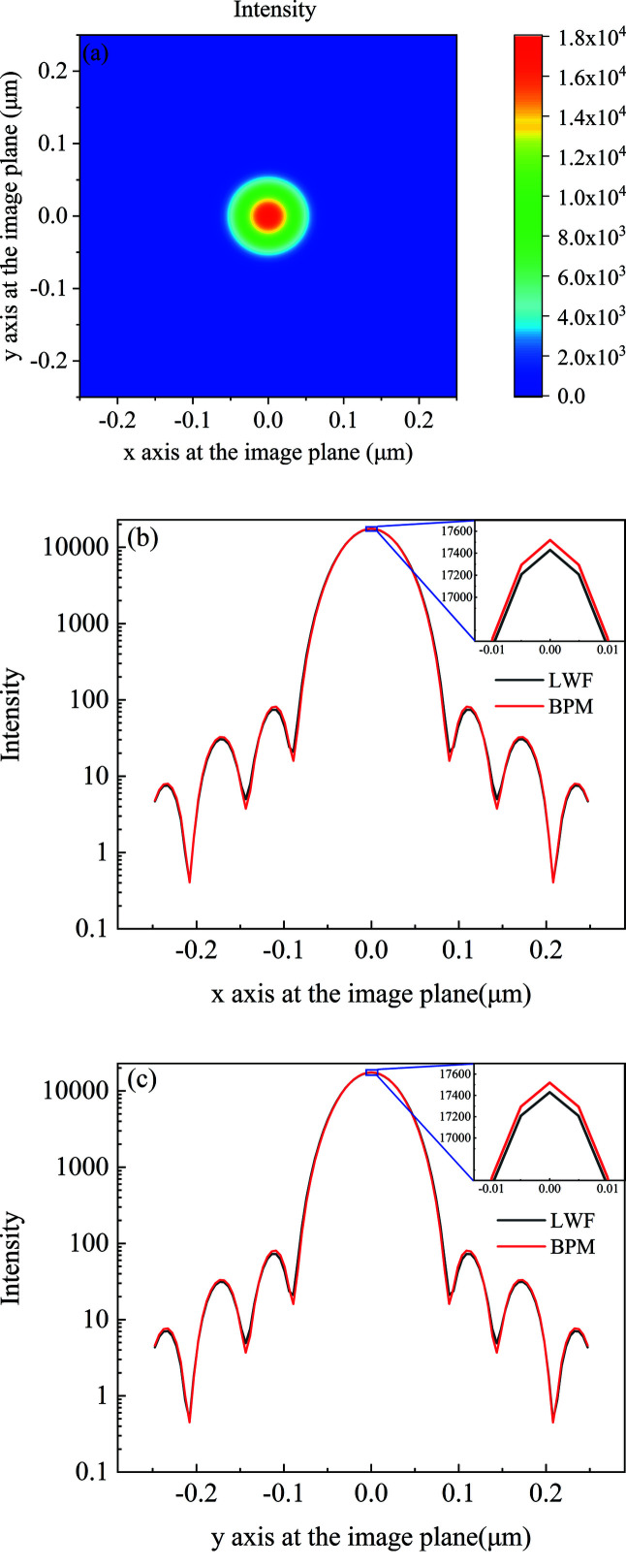
For the two-dimensional long kinoform lens, (*a*) the intensity distribution at the focal plane calculated from the LWF model, (*b*) the intensity distribution along the *x* axis at the focal plane calculated from the LWF model (black line) and BPM model (red line), and (*c*) the intensity distribution along the *y* axis at the focal plane calculated from the LWF model (black line) and BPM model (red line).

Due to the strong diffraction effect and multiple focal planes, the two-dimensional short kinoform lens is generally regarded as an improved structure of the zone plate (Petrov *et al.*, 2017 ▸; Sanli *et al.*, 2018 ▸). The long kinoform lens has only one focal plane and exhibits good refraction characteristics (Yan, 2010 ▸; Lin *et al.*, 2020 ▸). The two-dimensional long kinoform lens surface is generated by rotating the one-dimensional surface along the *z*-axis, and the parameters of the two-dimensional long kinoform lens are the same as those of the one-dimensional kinoform lens discussed in Section 3.1. The source energy is 11.3 keV with the complex amplitude of plane waves, and the light intensity has a Gaussian distribution with r.m.s. size of σ = 7 µm. The kinoform lens is located 20 m downstream of the light source. The aperture of the kinoform lens is 79.8 µm, and the total length of the lens is 4.594 mm with each step of length 229.7 µm and step number of 20. The incident surface is divided into 2000 × 2000 small elements with each element of size 40 nm × 40 nm. The intensity distribution in the focal plane is shown in Fig. 7 ▸(*a*). The spot size r.m.s. is 29.3 nm. The LWF model is used to benchmark against the BPM model. For the BPM model, the transversal wavefront is divided into 7000 × 7000 elements with dimensions of 11.4 nm × 11.4 nm. The number of wavefront slices perpendicular to the optical axis is 5000 with each slice of thickness 45.9 nm. As shown in Figs. 7 ▸(*a*) and 7 ▸(*b*), the intensity distribution from the BPM model (red line) is similar to the LWF result (black line), and the difference between the maximum intensities is less than 0.5%.

### 3.4. Calculation efficiency and accuracy

**Figure 8:**
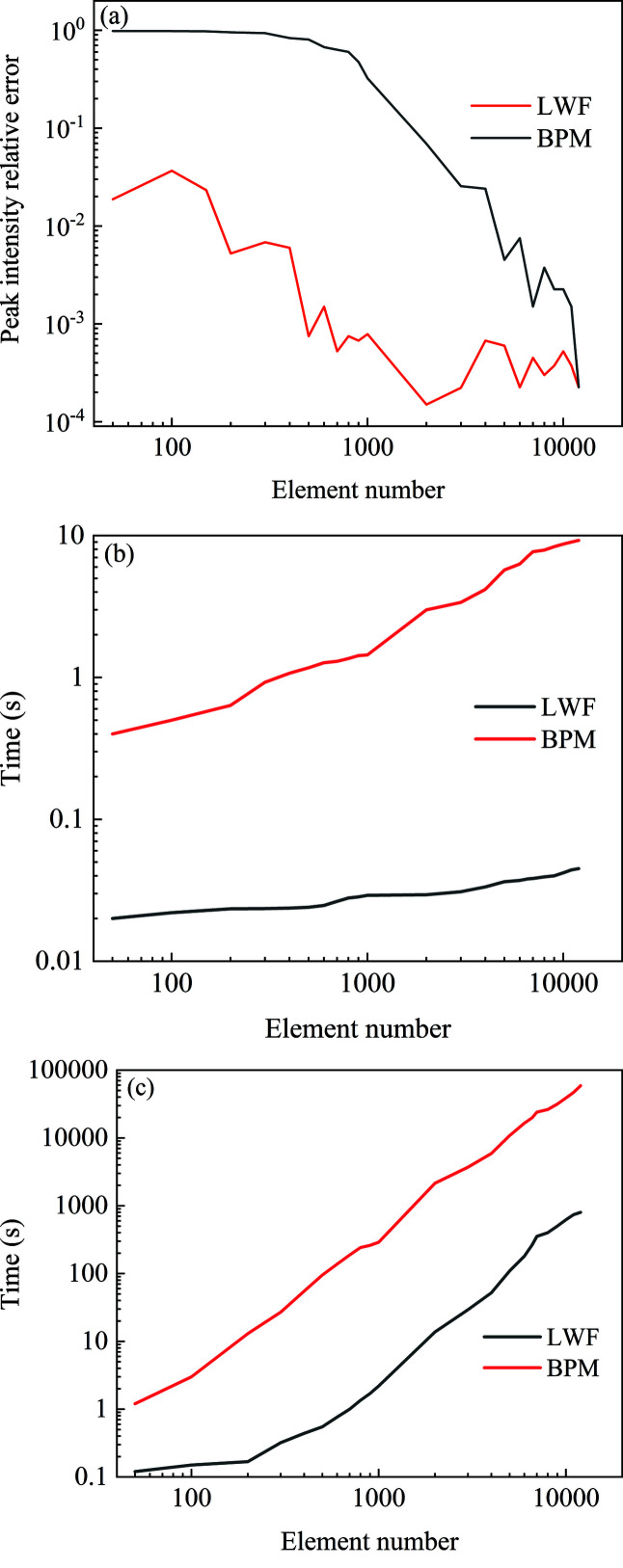
For the one-dimensional long kinoform lens, (*a*) the peak intensity relative error and (*b*) the calculation time as a function of element number. For the two-dimensional long kinoform lens, (*c*) the calculation time as a function of element number.

The LWF model is benchmarked against the BPM model, which has high calculation accuracy. Both the calculation accuracy and efficiency of the BPM model and the LWF model depend on the number of segments of the wavefront. In order to meet the approximate conditions of the BPM model, the wavefront slice thickness should be small enough to meet the assumption that the refractive index of the slice parallel to the optical axis is constant. The number of slices for the long and short kinoform lens is 5000 and 500, respectively. Under these cases, the calculation errors are less than 0.5%. We use the BPM model and the LWF model to calculate the maximum intensity at the focal plane for the one-dimensional long kinoform lens under different transversal division numbers. The optical parameters are the same as given in Section 3.1. With increase in the number of elements, the peak intensity for LWF and BPM tends to be constant. When the number of elements is greater than 10000, the peak intensity for the LWF model tends to be 133.4. The peak intensity relative error as a function of element number is shown in Fig. 8 ▸(*a*). Compared with the peak intensity for the element number of 10000, the calculation error is less than 0.5% for the LWF model (black line) when the transversal division number is 400. For the BPM model (red line), the calculation error is less than 0.5% when the element number is 7000. This indicates that the LWF model can obtain highly accurate results even with a small division number.

For the long kinoform lens, the calculation time as a function of transversal element number is shown in Fig. 8 ▸(*b*) for the one-dimensional lens and Fig. 8 ▸(*c*) for the two-dimensional lens. The two models use the same laptop computer with a single CPU core (i5-9300h, 2.4 GHz) and 16 GB RAM. For the LWF model, the number of elements should be 400 to achieve a calculation error of less than 0.5%. The corresponding calculation time is 0.025 s for the one-dimensional kinoform lens and is 5.3 s for the two-dimensional kinoform lens. For the BPM model, the number of elements should be 7000 to achieve a calculation error of less than 0.5%. The corresponding calculation time is 7.9 s for the one-dimensional kinoform lens and 24000 s for the two-dimensional kinoform lens. It can be seen that the LWF model has high computational efficiency, which is helpful for the optimization of kinoform lenses.

## 4. Conclusion

In this paper, based on wave optics and geometric optics, an LWF model is established to simulate X-ray propagation through a kinoform lens, including a long and a short kinoform lens. The intensity distribution at the focal plane from the LWF model is in good agreement with the BPM model. Since geometric optics are used to calculate X-ray propagation through the kinoform lens medium, the LWF model has high computational efficiency. Under 99.5% calculation accuracy, the calculation time for simulating X-ray propagation through the two-dimensional kinoform lens is only 5.3 s, which is helpful for the optimization of kinoform lenses.
